# Supplementary material for: Accurate prediction of substitution rates at protein sites with a mutation-selection model
Source: Sci Rep. 2025 Oct 2;15:34315. doi: 10.1038/s41598-025-22516-y (PMC12491638; doi:10.1038/s41598-025-22516-y)
Supplement: Supplementary file 1 — Supplementary Material 1 [file 41598_2025_22516_MOESM1_ESM.pdf]

# Accurate prediction of substitution rates at protein sites with a mutation-selection model

**Ingemar André**[1]

*[1]Biochemistry and Structural Biology, Lund University, PO BOX 124, Lund, Sweden*

*([ingemar.andre@biochemistry.lu.se](mailto:ingemar.andre@biochemistry.lu.se))*

# Supplementary Information

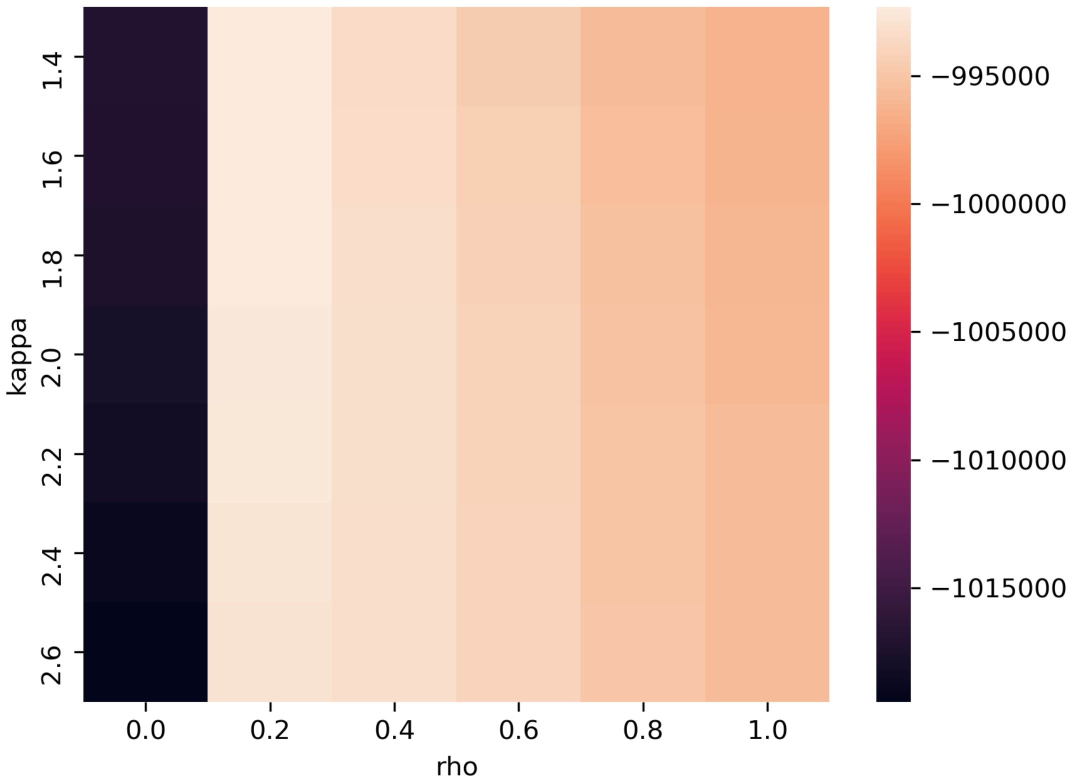


***Supplementary figure 1:*** *Maximum likelihood estimation of κ and ρ. Heatmap of summed likelihood for 59 alignments as function of κ and ρ.*

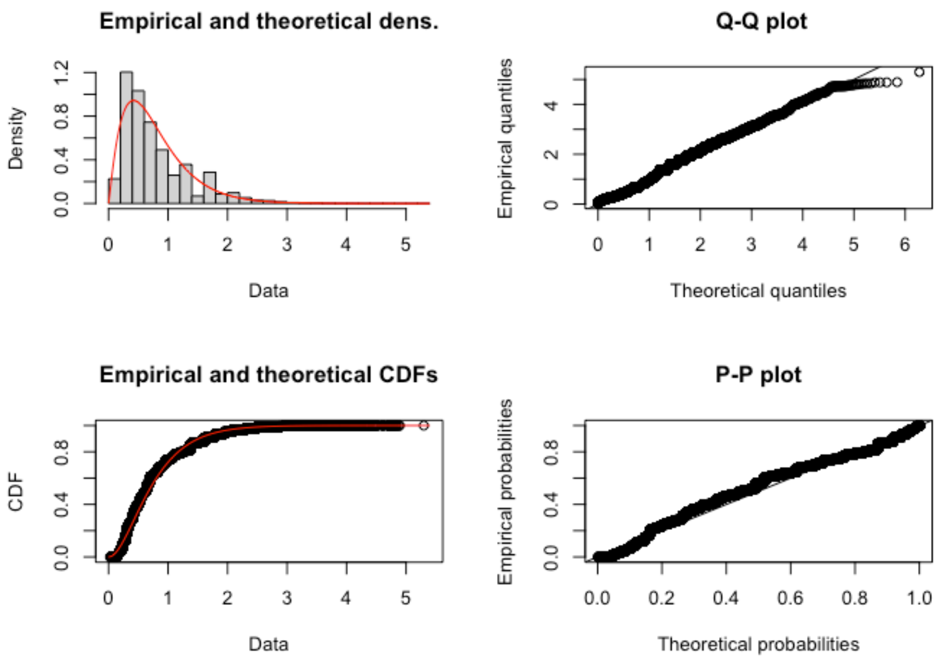


***Supplementary figure 2:*** *Fit of λ-distribution to a gamma function. Fitting result from fitdistr package in R(Team 2020) of the empirical histogram of λ-values from the multiple sequence alignments used to fit the LG substitution matrix.*

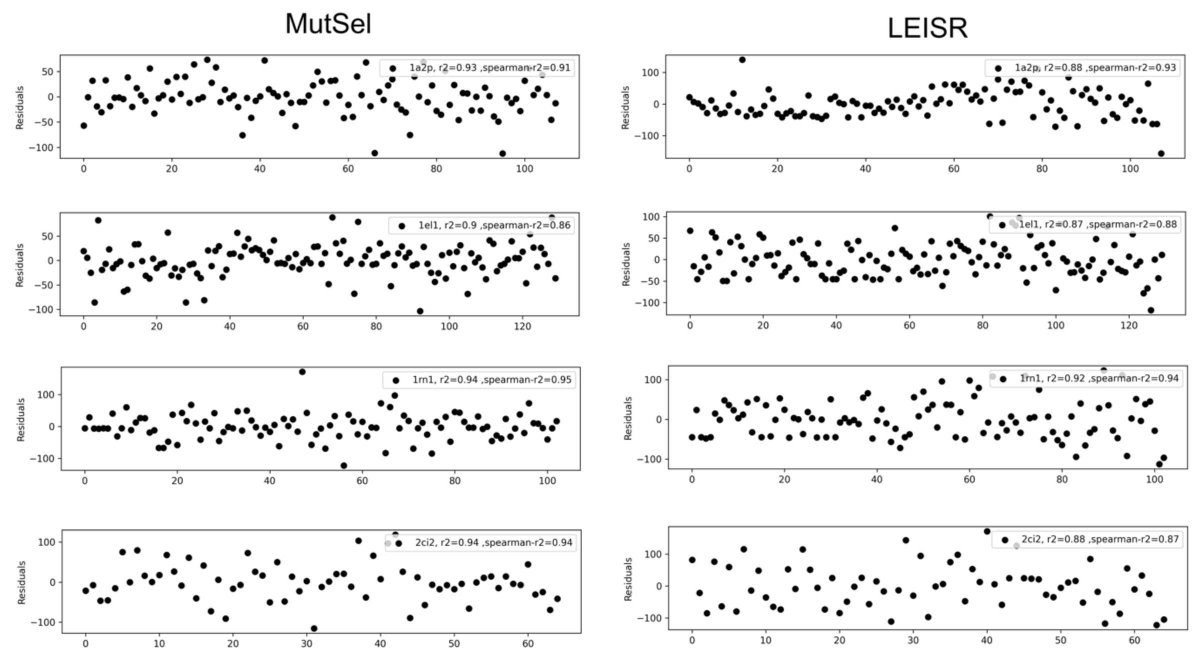


***Supplementary figure 3:*** *Fitting residuals and r2 and spearman r2 values corresponding to figure 2.*
